# The complete mitochondrial genome of *Grapsus tenuicrustatus* (Herbst, 1783) (Decapoda, Grapsidae)

**DOI:** 10.1080/23802359.2016.1180559

**Published:** 2016-07-08

**Authors:** Jin-Mo Sung, JeaHyun Lee, Sung-Keun Kim, Mustafa Zafer Karagozlu, Chang-Bae Kim

**Affiliations:** a Department of Life Science, Sangmyung University, Seoul, Korea;; b Bionics Co, Ltd, Seoul, Korea

**Keywords:** Arthropoda, complete mitochondrial genome, Decapoda, Grapsidae, *Grapsus tenuicrustatus*

## Abstract

A complete mitochondrial genome was sequenced from a grapsid crab, *Grapsus tenuicrustatus* (Herbst 1783), which was collected from a rocky intertidal zone of Chuuk lagoon. The size of mitochondrial genome is 15,858 bp with 31.9% A, 22.8% C, 12.2% G and 33.1% T distribution. Furthermore phylogenetic relationships of the Grapsoidea evaluated due to mitochondrial protein-coding genes. As per the obtained results, the families Grapsidae and Varunidae have sister group relationship in the superfamily Grapsoidea.

The grapsid crabs containing about 160 species in 9 genera inhabit rocky zones, estuaries, and marshes (WoRMS 2016). There is only one complete mitochondrial genome known from the Grapsidae species, *Pachygrapsus crassipes* (Yu et al. 2014). In this study, complete mitochondrial genome of grapsid species, *Grapsus tenuicrustatus* (Herbst 1783) sequenced and reported. This is the second record of complete mitochondrial genome from the family Grapsidae.

Specimens of *G. tenuicrustatus* were collected from the rocky intertidal zone of Weno Island, Chook Lagoon, Federated States of Micronesia (N 7°28′40″, E 151°53′49″) on 25 February 2015. The specimens were preserved in 97% ethanol and deposited in the Marine Biodiversity Institute of Korea (MABIK CR00235261).

The mitochondrial genome composed of 13 protein-coding genes, 2 ribosomal RNA genes 16S and 12S rRNA, and 22 tRNA genes. There are 10 overlapping regions in the genome with 1–8 bp length. The largest overlapping region is located between tRNA-Trp and tRNA-Cys. The genome shows 15 intergenic sequences varying from 1 to 48 bp in length. In comparison with *P. crassipes*, although they have identical gene order, their mitochondrial genome length is different. Size of mitochondrial genome of *G. tenuicrustatus* is 15,858 bp (GenBank accession number: KT878721), which is longer than *P. crassipes* mitochondrial genome (15,652 bp). The difference between two species can be explained by longer *NAD4* gene region of *G. tenuicrustatus*. While *G. tenuicrustatus* has 1,641 bp, *P. crassipes* has 1339 bp for same region. Another reason is the putative control region. *G. tenuicrustatus* have putative control region, 494 bp that located between 12S rRNA gene and tRNA-Ile in *G. tenuicrustatus* with 68.4% A–T content although *P. crassipes* does not have putative control region. Phylogenetic tree of Grapsoidea showed that *G. tenuicrustatus* and *P. crassipes* are members of the Grapsidae. Families Grapsidae and Varunidae have sister group relationship and Xenograpsidae early branched in the superfamily Grapsoidea (Figure 1). Similar results were observed in previous nuclear and mitochondrial gene-based molecular study (Tsang et al. 2014). This mitochondrial genome data provide genetic markers for phylogenetic of the grapsid crabs, which will be a part of mitochondrial genome library for provide evolutionary and systematic studies.

**Figure 1.**
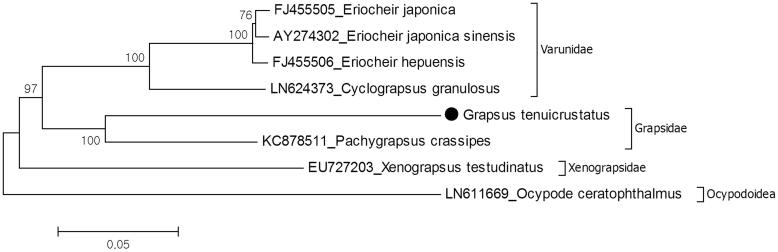
Molecular phylogeny of *G. tenuicrustatus* in the superfamily Grapsoidea. The phylogeny of G. tenuicrustatus reconstructed with maximum likelihood statistical method by MEGA 6 software (Tamura et al. 2013). For reconstruction, the complete mitochondrial genomes of the Grapsoidea species were retrieved from the GenBank and amino acid sequences of all protein coding genes except ATP8 gene were used for analysis. mtREV with Freqs (+F) model used for amino acid substitution and bootstrap method replicated 1000 times for the test of phylogeny. Ocypode ceratophthalmus which belongs to the superfamily Ocypodoidea chosen as representative of outgroup. For sequencing, the mitochondrial DNA was extracted from walking leg. Purified libraries were profiled using the Agilent Bioanalyzer and sequenced using the Illumina MiSeq platform to yield 300 bp paired end reads. Mitochondrial genes were assembled and annotated by MITObim software (Hahn et al. 2013) and MITOS web server (Bernt et al. 2013). The annotation of mitogenome sequences was refined by Geneious software version 9.1.2 (http://www.geneious.com, Kearse et al. 2012).
